# Study on the removal and transport and migration mechanism for As with activated sludge system

**DOI:** 10.1186/s13568-017-0478-y

**Published:** 2017-09-13

**Authors:** Jin Zhang, Wei Wei, Shuang Lin, Jie Lu, Qing Hu

**Affiliations:** School of Environmental Science and Engineering, Dalian Maritime University, Dalian, 116026 People’s Republic of China

**Keywords:** Activated sludge system, Transport mechanism, As removal, Physical and biological adsorption

## Abstract

In this paper, the removal of As and the transport and migration of As at the process of activated sludge system was studied. The results showed that the activated sludge system has high removal efficiency for As, and the removal efficiency could be nearly 100%. The initial concentration of As has effect on the removal efficiency, and within the experimental scope, the increase of initial concentration of As improved the removal efficiency for As. The distribution of As in the surface and internal in activated sludge indicates that in the process of activated sludge, the As was first transferred from water to the surface of solid and was adsorbed by the surface and then transport to the interior of microbes and take part in some metabolisms of the microorganisms. The adsorption of As by activated sludge conforms to Freundlich adsorption isotherm, and the removal of As in activated sludge system follows to the pseudo second order reaction kinetics.

## Introduction

With the development of industry and agriculture, there are more and more heavy metal pollution in environment. As one kind of heavy metal, arsenic (As) has caused serious influence on water environment. By estimated, the total amount of As flow into the water environment is about 110 thousand tons per year (Bansod et al. 2017; Zheng et al. 2017; Mukherjee et al. 2017).

Arsenic and its compounds are carcinogenic to humans and other species, and are considered to be as one of environmental virulence metal together with cadmium, lead, mercury and chromium (Shen et al. 2017). Once arsenic enters into the human body, the skin, the cardiovascular systems, the nervous system, the kidney and the liver will be injured significantly. When the concentration of arsenic in the human body is 0.01–0.052 g, it will lead to arsenic poisoning; when the concentration of arsenic is 0.06–0.2 g, it will lead to death (Zeng et al. 2017; Nandre et al. 2017; Mukherjee et al. 2017; Kempahanumakkagari et al. 2017). Arsenic was considered as the priority controlled pollutants by World Health Organization (WHO) (El-Moselhy et al. 2017; Miroslav et al. 2016). In April 5, 2009, the United Nations Children’s Fund Published news bulletins in Dhaka, saying that there are 60 million people around the world are drinking water polluted by arsenic, and about 80% of them lived in Asia. With the rapid development of society, the use of arsenic is bound to expand, and the wastewater containing arsenic increases greatly. Therefore, the removal for arsenic has becoming the focus problem in water and wastewater treatment (Ding et al. 2017; Molinari and Argurio 2017; Bhowmick et al. 2015).

Biological treatment technology is developed rapidly and has been used widely in wastewater treatment (Wang et al. 2017; Erkan et al. 2017; Chen et al. 2017). Activated sludge process is one kind of biological treatment technology, and mainly focused on removing BOD or COD. Biodegradable organic matter (BOM) provides substrate for microbial re-growth in the distribution system, biological processes can effectively remove organic matter and ammonia content from raw water. In system of activated sludge technology, the microorganisms exit in form of activated sludge, which could absorb and degrade pollution matters in sewage, and result in the decrease of many kinds of pollution (Mujtaba and Lee 2017). Because of high efficiency and strong adaptability, activated sludge technology has been seemed as the main technology in sewage and wastewater treatment. Many heavy metals have toxic on microorganisms. While in treatment system of activated sludge, some metals with low concentration are usually absorbed by activated sludge, and reduced the metals from water and wastewater.

In this paper, the effect of activated sludge system in As removal was studied, and the mechanism of As removal in activated sludge system has been explored.

## Materials and methods

### The main experimental materials

Inoculation sludge was obtained from the feedback sludge tank of the municipal wastewater treatment plant located in Lingshui of Dalian and pre–incubated before being put into the reactor. Synthetic wastewater fed to the reactor consisted of sucrose, NH_4_Cl, K_2_HPO_4_, KH_2_PO_4_ and mineral solution (containing MgSO_4_·7H_2_O, CaCl_2_·2H_2_O, FeSO_4_·2H_2_O, CoCl). The experiment was carried out in a SBR reactor, and the effective volume of the reactor is 2 L. All reagents used were pure analysis and purchased from China. The phosphate buffer solution (pH:5.8) was purchased from Duanfeng biotechnology co. LTD, China. The As standard liquid (1000 μg/mL) purchased from TMRM Standard material center, China was used to prepare the wastewater contain As.

### Analysis method

As concentration was analyzed with atomic fluorescence spectrometry. Based on the characteristics of activated sludge, the As will be absorbed onto the surface of activated sludge first, then may be migrated to the interior of the microorganisms and take part in the metabolisms of microorganisms. Thus, the As removed from water may be caused by the follow steps: some of As may be removed only by the surface adsorption of sludge, some may be removed by interior absorb of the microorganisms. In order to explore the mechanism of As removal by the activated sludge, the As content in microbial sludge was analyzed. The suspension of activated sludge was treated for the analysis of As to analyze the mechanism of As removal, and the treatment method of the suspension of activated sludge was described as follow.

### Treatment for the suspension of activated sludge

The suspension of activated sludge of 20 mL was centrifuged at 4000 rpm for 10 min. The supernatant was used to analyze the arsenic content and seemed as the residual As content in the water samples (C_L_) and the removal efficiency of As from water was calculated as the following Eq. (1); the settled sludge was further processed for analysis of As content in solid status of sludge.

1 $$\text{Re} moval = \frac{{C_{\text{L0}} - C_{L} }}{{C_{L0} }}$$

where C_L0_ is the initial concentration of As in water.

### Treatment for the settled sludge

10 mL of phosphate buffer solution was added to the settled sludge and shake to disperse the sludge evenly for 10 min, and then the mixed suspension was centrifuged at 4000 rpm for 10 min. The supernatant and the settled sludge were separated and 10 mL of deionized water was added to the settled sludge and was centrifuged again as the same manner. The supernatant of the two steps was mixed together and the As content was analyzed and seemed as the As content adsorbed by the surface of activated sludge (C_SS_). The residual sludge was processed again as method as following and to analyze the As content and seemed as the As absorbed into the interior of microorganisms (C_IS_).

### Treatment for the residual sludge

The residual sludge was drying at 85 °C for 3 h and then grind to power less than 0.15 mm for the subsequent digestion. 0.02 g of the power was mixed with 10 ml aqua regia (HNO_3_:HCL = 1:1) and digestion for 2 h in boiling water bath and shake once every half an hour. After cooling, the As content in supernatant was analyzed and seemed as C_IS_.

## Results

### Removal for As with activated sludge system

**Fig. 1:**
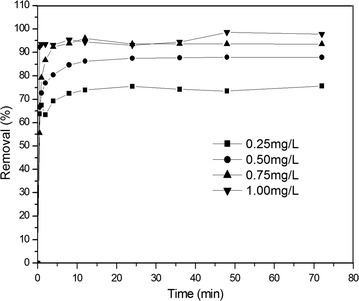
The removal efficiency for As with the activated sludge system

The activated sludge system was used to treat water contaminated with As. The concentration of activated sludge was 2500 mg/L, and the initial concentration of As in water was controlled at 0.25, 0.50, 0.75 and 1.00 mg/L respectively. The removal efficiency for As with the activated sludge system was shown in Fig. 1.

From the results in Fig. 1, it could be seen that the removal for As by the activated sludge was very fast. The removal efficiency increased with the process and reached stable after a short time of the process, which indicated that the As was not only adsorbed by the surface of activated sludge, but also was transported to the interior of the microorganisms and take part in the metabolic processes of the microorganisms (Rashid et al. 2016; Kilic et al. 2008; Martínez-Alcalá et al. 2017). At the same time, the removal of As increased slightly with the increase of the initial concentration of As. Calculated results showed that when the initial concentration of As was 0.25 mg/L, the maximum removal of As by activated sludge was 0.06, 0.17, 0.28 and 0.39 mg/g, respectively. When the initial concentration of As was 0.5 mg/L, the maximum removal amount was 0.17 mg/g. When the initial concentration of As was 0.75 mg/L, the maximum removal amount was 0.28 mg/g. When the initial concentration of As was 1.00 mg/L, the maximum removal amount reached 0.39 mg/g. When the initial concentration of As is high, there is a large gradient difference in As concentration between water and the surface of sludge particles, which increases the driving force for mass transfer and enhance the removal efficiency for As.

### Kinetics and thermodynamics of removing arsenic by activated sludge system

#### The reaction kinetics study

**Fig. 2:**
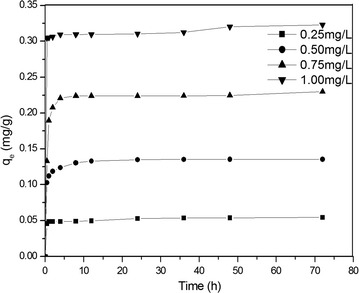
Removal kinetics for As with activated sludge

The initial concentration of As was controlled at 0.25, 0.50, 0.75 and 1.00 mg/L. The concentration of activated sludge was 2500 mg/L. According to formula (2), the removal amount at different time was analysis, and the results were shown in Fig. 2.

2 $$q_{t} = \frac{{V(C_{\text{L0}} - C_{L} )}}{m}$$

In which, *q*_*t*_ is removal amount (mg/g); V is the volume of the solution (L); *C*_*L*0_ is the initial concentration of As (mg/L), and *C*_*L*_ is the concentration of As in water at time of *t*; *m* is the mass of the activated sludge (g).

As shown in Fig. 2, the removal amount increased quickly in the first 0.5–2.0 h, and then increased slowly to stable level.

The removal kinetics data were fitted according to pseudo first order kinetics model and pseudo second order kinetics model according to the formulas (3) and (4). The fitted results were shown in Table 1.

**Table 1:** Kinetic parameters of removal for As

Initial concentration of As (mg/L)	q_e_ (Experimental results)	Pseudo first order kinetics			Pseudo second order kinetics		
		q_e_	k_1_	R^2^	q_e_	k_2_	R^2^
0.25	0.054	0.010	0.062	0.730	0.054	40.281	1.000
0.50	0.135	0.030	0.149	0.883	0.136	45.187	0.995
0.75	0.229	0.032	0.149	0.413	0.229	15.985	1.000
0.10	0.323	0.030	0.045	0.417	0.322	11.545	1.000

3 $$\ln (q_{e} - q_{t} ) = \ln q_{e1} - k_{1} t$$

4 $$\frac{t}{{q_{t} }} = \frac{1}{{k_{2} q_{e2}^{2} }} + \frac{t}{{q_{e2} }}$$

In which: *q*_*t*_(mg/g) is the removal amount at time *t*; *q*_*e*_ (mg/g) is the removal equilibrium amount; t(min) is the contact time; *k*_1_ and *k*_2_ is rate constant of the first and second order kinetics respectively.

From the results in Table 1, it could be seen that the R^2^ of the second order kinetics model of the four conditions is nearly to 1.000 and is obviously superior to the first order kinetics model, which indicates that the removal of arsenic with the activated sludge conforms to the second order kinetics model.

#### Study on thermodynamic of As removal in activated sludge system

In activated sludge system, the removal of As may be contributed mainly to the surface adsorption of activated sludge. The adsorption isotherms were studied to explore the mechanism of surface adsorption. The concentration of activated sludge was 2500 mg/L, and the initial concentration of As was controlled at 0.2, 0.3, 0.4, 0.5, 0.6, 1.0, 1.2, 1.5, 2.0, 3.0, 5.0, 10.0, 20.0 and 50.0 mg/L respectively. The contact time was 24 h. The analysis results of Langmuir adsorption isotherm (5) and Freundlich adsorption isotherm (6) were shown in Table 2.

5 $$q_{c} = \frac{{aK_{L} C_{e} }}{{1 + K_{L} C_{e} }}$$

6 $$q_{c} = K_{F} C_{e}^{n}$$

**Table 2:** Parameters of Langmuir and Freundlich Equations

Langmuir equation			Freundlich equation		
a	K_L_	R^2^	n	K_F_	R^2^
27.778	0.120	0.877	2.767	0.901	0.968

The fitted results showed that the surface adsorption of activated sludge for As can be described well with Freundlich adsorption isotherms very well.

#### The migration and transport mechanism of arsenic in the activated sludge system

The activated sludge reactor was conducted at continuous flow mode, and the HRT was controlled at 12 h. The initial concentration of As was 0.25 mg/L.

**Fig. 3:**
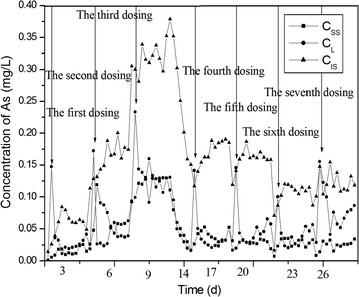
The distribution of As in continuous flow activated sludge reactor. *CL* concentration of As in water, *CSS* concentration of As in the surface of solid, *CIS* concentration of As in the interior of solid

**Fig. 4:**
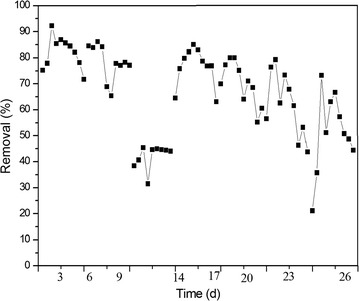
The removal of As in continuous operation mode

The concentration of As was analyzed according method described in 2.2. The distribution of As was shown in Fig. 3, and the total removal of As was shown in Fig. 4.

It could be seen from Fig. 4 that in the whole process, the removal rate for As kept at a higher level of 70–80%. But as shown in Fig. 3, there exist some increase of As content in the third stage. Then the addition of As was stop in the experiment, and the activated sludge was cultivated without the high contamination of As for about 5 days and then to continue the experiment. But in over all, the removal of As declined gradually with the long process shown in Fig. 4.

As shown in Fig. 3, the As content in water decreased quickly at the first day of every stage, and then kept at the low level. But the As content adsorbed in surface of activated sludge was kept at a lower level all the time. While the As content in the interior of microorganisms increase at the beginning in every stage.

## Discussion

**Fig. 5:**
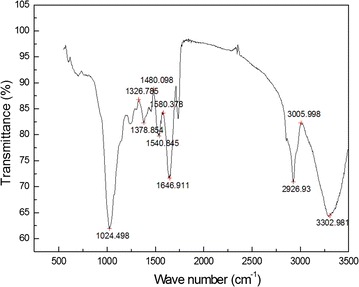
The FT IR spectrum of activated sludge

Because of many surface functional groups and characteristics of adsorption of activated sludge, the activated sludge system has high performance for the removal of As. There are mainly the characteristic peaks in FT IR spectrum (Fig. 5) of the stretching vibration of –OH of alcohols, acids and acid-like substances on the surface of activated sludge (Lee et al. 2013). The stretching vibration peak in the range of 2930–2925 cm^−1^ (Liu et al. 2012; Zhao et al. 2015) is the –CH peak. In the range of about 1640–1660 cm^−1^ (Song et al. 2014), the stretching vibration peaks are the peaks of –C=O and –CN on the protein. At around 1250 cm^−1^ (Yan et al. 2010), the peak is the –C=O deformation peak of the nucleic acid. At 1024 cm^−1^, the peak is the stretching vibration peak of –OH in polysaccharide. The peaks at below 1000 cm^−1^ are the fingerprints of phosphate functional groups, sulfur functional groups, etc. (Aravindhan et al. 2004). Large number of surface functional groups contribute to the removal of As.

The As was absorbed in the surface of activated sludge first, and then was transferred into the interior of microorganisms and take part in the metabolic process of microorganisms. The mass distribution of arsenic in solid and liquid phase in the activated sludge system varies with time (Table 3). The distribution trend of arsenic is that the As was gradual migrate from liquid to solid. In the first 0.5 h, the migration rate was very high, and then down slowly. This indicates that the As was mainly adsorbed by the activated sludge in the first 0.5 h. The Distribution Factors (DF) increased with the increase of the initial concentration. The reason may be that when concentration of arsenic is low, the amount of As absorbed by microorganisms is low, while with the increase of concentration of As, there are more arsenic absorbed by microorganisms.

**Table 3:** DF of As in liquid phase and solid phase (%)

Initial concentration	0.1 h	0.5 h	1 h	2 h	4 h	8 h	12 h	24 h
0.25 mg/L								
DF_L_	80.2	34.9	31.9	34.3	41.1	31.8	21.2	23.9
DF_S_	19.8	65.1	68.1	65.7	58.9	68.2	78.8	76.1
0.50 mg/L								
DF_L_	74.1	41.1	38.3	30.8	18.3	13.2	19.7	23.4
DF_S_	25.9	58.9	61.7	69.2	81.7	86.8	80.3	76.6
0.75 mg/L								
DF_L_	79.8	57.4	32.6	15.4	9.5	6.4	5.7	11.0
DF_S_	20.2	42.6	67.4	84.6	90.5	93.6	94.3	89.0
1.00 mg/L								
DF_L_	78.6	54.6	34.2	22.1	9.9	8.3	4.3	7.5
DF_S_	21.4	45.4	65.8	77.9	90.1	91.7	95.7	92.5

In which, DF_L/S_ distribution factors, and C_L_ is the amount of As dissolved in the liquid phase, C_S_ is the amount of As adsorbed in the solid phase, C_T_ is the total amount of As in the liquid and solid.

7 $${\text{DF}}_{L} = \frac{{C_{L} }}{{C_{T} }}$$

8 $${\text{DF}}_{S} = \frac{{C_{S} }}{{C_{T} }}$$

**Fig. 6:**
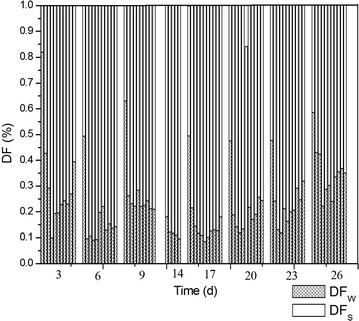
Distribution of As between fluid phase and solid phase

The DF_W_ increased at the first day of every stage and then decreased in the continuous flow mode (Fig. 6). These are because that the As was first adsorbed by the surface of activated sludge and then transferred into the interior of microorganisms and participate in the metabolic activity, and the rate of metabolism was lower and there exist some accumulation of As in the interior of microorganisms. With the increase of As content accumulated in the interior of the microbes, the microbial metabolism will be inhibited to a certain extent.

The activated sludge system has high removal efficiency for As in water. In scope of low initial concentration of As in the study, the removal efficiency increase with the increase of the initial concentration of As. The As in water was first adsorbed in the surface of activated sludge, and then some was transport and migrated to the interior of microbes and take part in the metabolic activity of the microbes.

## Abbreviation

As: arsenic
